# Ficolin-2 Lectin Complement Pathway Mediates Capsule-Specific Innate Immunity Against Invasive Pneumococcal Disease

**DOI:** 10.3389/fimmu.2022.841062

**Published:** 2022-03-28

**Authors:** Moon H. Nahm, Jigui Yu, Juan J. Calix, Feroze Ganaie

**Affiliations:** ^1^ Division of Pulmonary, Allergy, and Critical Care Medicine, Department of Medicine, Heersink School of Medicine, University of Alabama at Birmingham, Birmingham, AL, United States; ^2^ Division of Infectious Diseases, Department of Medicine, Heersink School of Medicine, University of Alabama at Birmingham, Birmingham, AL, United States

**Keywords:** lectin complement pathway, ficolin-2, *Streptococcus pneumoniae*, innate immunity, capsule

## Abstract

Reports conflict regarding which lectin-microbial ligand interactions elicit a protective response from the lectin pathway (LP) of complement. Using fluorescent microscopy, we demonstrate the human lectin ficolin-2 binds to *Streptococcus pneumoniae* serotype 11A capsule polysaccharide dependent on the O-acetyltransferase gene *wcjE*. This triggers complement deposition and promotes opsonophagocytosis of encapsulated pneumococci. Even partial loss of ficolin-2 ligand expression through *wcjE* mutation abrogated bacterial killing. Ficolin-2 did not interact with any pneumococcal non-capsule structures, including teichoic acid. We describe multiple 11A clonal derivatives expressing varying degrees of *wcjE*-dependent epitopes co-isolated from single blood specimens, likely representing microevolutionary shifts towards *wcjE*-deficient populations during invasive pneumococcal disease (IPD). We find epidemiological evidence of *wcjE* impairing pneumococcal invasiveness, supporting that the LP’s ficolin-2 axis provides innate, serotype-specific serological protection against IPD. The fact that the LP is triggered by only a few discrete carbohydrate ligands emphasizes the need to reevaluate its impact in a glycopolymer-specific manner.

## Introduction

The complement system is a series of interconnected enzymatic cascades that when triggered, mediate inflammatory signaling, the formation of surface effector complexes, and phagocyte recruitment (1), ultimately promoting the clearance of microbes or damaged host cells. While all cascades converge with complement component C3b covalently opsonizing target surfaces, three principal pathways are defined according to how each is triggered. The alternative pathway is continually triggered at low levels by spontaneous hydrolysis of ubiquitous proenzymes. Conversely, the classical pathway and lectin pathway (LP) are triggered when pathway-specific recognition molecule/enzyme activator complexes interact with surface-associated immunoglobulin or carbohydrate ligands, respectively. The non-specificity of the alternative pathway and the fact that classical pathway targets span antigens recognizable by the adaptive immune system, explain in part why individuals with deficiencies in these two pathways experience increased risk to various infections (2–5).

In contrast, LP targets are limited to the ligands of a few host-specific [six identified in humans (6)] soluble lectins. Despite being triggered by a fixed range of targets, *in vitro* and animal studies suggest that the LP provides innate protection against prevalent Gram-positive bacterial pathogens (7–9). For example, ficolin-2 (also called L-ficolin) is the second most abundant LP lectin activator in human serum (6) and is widely reported to recognize the teichoic acid (TA) and lipoteichoic acid (LTA) surface glycopolymers of multiple bacterial species (10, 11). Notably, Vassal-Stermann et al. reported the LP is triggered by ficolin-2 binding to phosphocholine-dependent TA/LTA structures characteristically present on the surface of the major pathobiont *Streptococcus pneumoniae* (the pneumococcus) (12), suggesting that the LP ficolin-2 axis confers broad protection against pneumococcal disease. However, no link has been consistently detected between LP deficiencies and pneumococcal infection risk in humans, even though up to 1 in 1000 individuals display deficient production of mannose-binding lectin-associated serine protease-2 (MASP-2), the principal LP enzyme activator (13, 14). Thus, under the presumption that the LP is capable of broadly targeting various infectious agents, it is deemed redundant in protecting against infection, and its association with any immune deficiency remains controversial (15, 16).

Contrary to reports of the LP being triggered by ubiquitous glycopolymers like TA/LTA, others have shown that the LP, and specifically the ficolin-2 axis, is *effectively* triggered by ligands exclusively expressed by only subsets of a microbial species. For example, ficolin-2 binds pneumococci expressing serotype 11A capsule polysaccharide (PS) and mediates antibody-independent deposition of accessible complement components C3b and C4b on the bacterial surface, which in turn promotes opsonophagocytosis (17, 18). However, ficolin-2 does not bind serotype 11E capsule, a capsule type derived from 11A following inactivation of the capsule O-acetyltransferase gene *wcjE* and resultant loss of 11A PS-defining β-galactose-6-O-acetylation (19, 20). Indeed, ficolin-2 binds almost all capsule types containing *wcjE*-mediated O-acetylation, with a notable exception being that it does not bind the *wcjE*-dependent serotype 9V nor its *wcjE*-null counterpart 9A (17, 18). If indeed the LP is effective only against a subset of serotypes, disease caused by other prevalent serotypes could conceivably mask the LP’s protective impact at the population level.

Here we sought to resolve discrepant findings regarding how the LP ficolin-2 axis interacts with pneumococci. We also report and contextualize *in vitro*, epidemiological and clinical isolate whole genome sequencing findings that demonstrate how the LP provides serotype-specific innate protection against invasive pneumococcal disease (IPD) at the population level and, thus, make a case for the need to reevaluate the role of the LP in human health.

## Methods

### Reagents and Buffer Preparation

Reagents were purchased from Sigma Aldrich (St. Louis, USA) or Thermo Fisher Scientific (Waltham, USA) unless otherwise specified. Factor 11c antiserum (Statens Serum Institut, Copenhagen, Denmark) was used to detect serotype 11A, 11E and 11D capsule (20). Hybridoma supernatants containing Hyp11AG2 and Hyp9VG2 murine monoclonal antibodies (mAb), were used for detection of serotype 11A and 9V capsule, respectively, as previously described (21, 22). Hanks’ buffered saline solution (HBSS) plus calcium buffer (HBC) was HBSS supplemented with 2.2 mM CaCl2 and 0.5% bovine serum albumin (BSA). Opsonization buffer (OBB) was HBBS containing Ca2+/Mg2+ supplemented with 0.1% gelatin and 5.2% fetal bovine serum. Cell expression media (CEM) was Dulbecco’s Modified Eagle’s Medium/Nutrient Mixture F12 supplemented with 10% heat-inactivated fetal bovine serum (FBS) and 750 μg/mL of geneticin (Life Technologies, Carlsbad, USA).

### Complement Sources and Inhibitors

Normal human serum (NHS) was obtained from a healthy adult volunteer donor according to a protocol approved by the University of Alabama at Birmingham Institutional Review Board (IRB-140618001). For a complement source lacking classical pathway activity, we used commercially available C1q-depleted human serum (Complement Technology, Inc., Texas, USA; A300), which we have shown to also be depleted of ficolin-2 (ΔC1f2 serum) (23). Affinity-purified recombinant ficolin-2 (rFicolin-2) or CEM supernatant containing 1.2mg/L rFicolin-2 (CEMf2), was obtained from huf2E, a Chinese Hamster Ovary K1 cell line transfected with the human FCN2 gene, as previously described (24). In inhibition experiments, preparations of ΔC1f2 serum with rFicolin-2 were preincubated on ice for 60 min alone or with the following final concentrations of inhibitors: 10 mg/L acetylated BSA (Sigma Aldrich, St. Louis, USA), 10 mg/L BSA (Sigma Aldrich, St. Louis, USA), 100 mg/L 11A capsule polysaccharide (American Type Culture Collection, Manassas, USA), 100 mg/L of cell wall polysaccharide/teichoic acid (Staten Serum Institut, Copenhagen Denmark), or 1:100 diluted silica clot activator (SCA) obtained through elution of a 10-ml plastic red-top tube (catalog no. 367820; BD Biosciences, Franklin Lakes, USA) with 1 ml of water, as previously described (23).

### Strains and Culture Conditions

The strains used in this study are listed in **Table 1**. Clinical isolate 4011-06 [obtained from the USA Centers for Disease Control and Prevention (CDC) as a “serotype 11A” specimen (19, 27)] contained the three strains MNY31, MNY32, and MNY33, which were identified and subcloned according to their distinct degrees of reactivity with Hyp11AG2 mAb (21). The clinical strains AP278, MNZ2293-A, and MNZ2293-C were characterized previously (21, 25). The laboratory and recombinant strains SPEC6B, MNZ272, JC03, JC04, AMB03, and TIGR-JS were described previously (19, 21, 28). Reference strains TIGR4, D39, and R36A were kindly provided by David Briles (Birmingham, USA). Unless otherwise noted, bacteria were cultured at 37°C and 5% CO2 on blood agar plates (BD Biosciences, Franklin Lakes, USA) or in Todd Hewitt broth or agar (BD Biosciences, Franklin Lakes, USA) with 0.5% yeast extract (THY). Freezer stocks were made from THY broth cultures (OD600 = 0.6) supplemented with 15% glycerol and stored at -80°C until needed.

**Table 1:** *S. pneumoniae* strains and primers used in this study.

Strain	Serotype	Genotype	Ficolin-2 binding^c^	Reference
Clinical strains				
MNZ272	11A	WT	++	(19)
MNY31	11A	WT	++	current study
MNY32	11E	MNY31, *wcjE* 501::ISSpn5	–	current study
MNY33	11A variant	MNY31, *wcjE* 969::IS1515	–	current study
AP278	11A variant	*wcjE* 968::IS1515	+	(25)
MNZ2293-A	11A	WT	++	(26)
MNZ2293-C	11A variant	MNZ2293-A, *wcjE* C502T	+	(26)
TIGR4	4	WT	–	
D39	2	WT	–	
SPEC6B	6B	derived from WT	–	
				
Recombinant strains				
JC03	11A	MNZ270, *rpsL*::*rpsL*_TIGR_	++	(21)
JC04	11E	JC03, *wcjE*::JS^a^	–	(21)
OC6.8	11E/11A	JC04, P_tet_-*wcjE*_11A_ ^b^	+	current study
JC02	9A	JC01[9V], *wcjE*::JS^a^	–	(22)
FG02	9A/9V	JC02, P_tet_-*wcjE*_11A_ ^b^	–	current study
R36A	none		–	
TIGR4-JS	none	TIGR4, *cps* locus::JS^a^	–	
AMB03	none	JC03, *wchA,wchJ,wchK,wcyK,wcwC,wcrL*::JS^a^	–	(17)
**Primer Name**		**Sequence**		
5964		ctttagtcaactagagcaaggatctgcatcatact		
3299		aaattaaaataacttgtgagcttggactagaaaaaaacttcac		
5965		ccttgctctagttgactaaagtaagaattaattgggtagattttgggaaaggat		
3300		caagctcacaagttattttaatttaatatacttttgtggtaataaaacaatattcaaaaa		
51092		ATTTTGAAatgactaaagtaagaattaattgggtagattttggg		
3925		GCCTCCTTActagagcaaggatctgcatcatactctatc		

a , Recombinatorial replacement of entire gene(s) with Janus cassette (JS).

b , Cassette containing tmp,Ptet-wcjE, and tetR inserted between genomic bgaA and spr0566-spr0568.

c , ‘++’, strong binding, ‘+’, low binding, ‘-’, no binding; WT, wild type.

### Construction of Pneumococcal Strains, OC6.8 and FG02

Primers used in this study are listed in **Table 1**. Plasmid pTEX2 was kindly provided by Reinhold Bruckner (Nürnberg, Germany) and contains a cargo region under control of Tet-On inducible promoter (29). Plasmid pONC6.8 was obtained by fusing pTEX2 linearized without its cargo (PCR amplified with primers 5964 and 3299) and a 1052 bp DNA fragment containing *wcjE* (PCR amplified with primers 5965 and 3300 using MNZ272 genomic DNA as template), using HiFi DNA assembly per manufacturer recommendations (New England Biolabs, Ipswich, USA); and then performing site-directed mutagenesis using primer 51092 and 3925 to replace the native *wcjE* TTG start codon with a TAAGGAGGCATTTGA**ATG** ribosomal binding site (underlined) plus ATG start codon (bolded). Recombinant strains OC6.8 and FG02 were obtained by transforming pONC6.8 into the serotype 11E strain JC04 and serotype 9A strain JC02, respectively, with selection on THY agar containing 15μg/mL trimethoprim. This resulted in insertion of cargo DNA adjacent to the genomic *bgaA* site. Sequence of insert introduced 0C6.8 is available on NCBI under accession number MZ054181. Expression of *wcjE* was induced by incubating bacteria in THY broth containing 100µg/mL anhydrous tetracycline (Abcam, Cambridge, UK) at 37°C for 30 min.

### Visualizing Bacterial Surface Binding Using Fluorescent Microscopy

Bacterial freezer stocks were thawed, washed twice, and resuspended to an OD_600_ = 0.5 in HBC. Suspended bacteria were then mixed 1:1 with HBC containing either 10% NHS, 0.1% factor 11c antiserum, 1% Hyp11AG2 or Hyp9VG2 supernatant, or 20 µg/mL of biotin-conjugated Dolichos biflorus agglutinin (DBA, Vector Laboratories, Inc., Burlingame, USA), which detects the Forsmann antigen on pneumococcal LTA/TA (30), and then incubated on ice for 60 min. Bacteria were washed and resuspended in HBC containing fluourescein-labeled anti-ficolin-2 antibody, anti-rabbit antibody, anti-mouse IgG antibody, or streptavidin, respectively, and incubated in the dark on ice for 30 min. Bacteria were washed, suspended in 200µL of PBS with 5µM of DRAQ5, and incubated at room temperature for 30 minutes. Bacteria were washed and suspended in 20µL of 10% neutral buffered formalin solution (Sigma-Aldrich, St. Louis, USA). Bacteria were deposited and allowed to settle on a coverslip for 30 min at RT. After gently washing unbound bacteria with PBS, coverslips were placed onto 20µL of Fluoromount G (Southern Biotech, Birmingham, USA) deposited on a microscope slide, incubated at 4°C for 24 h, and sealed with nail polish. Specimens were visualized on an A1R SIM fluorescence microscope (Nikon, Tokyo, Japan) with 1x100 oil objective and 1.45 numerical aperture, and captured with a Hamamatsu SIM camera. 3D reconstitutions, deconvolution and gamma adjustments were performed using Element software (Nikon, Tokyo, Japan).

### Detection of Complement Deposition on Bacterial Surface

Gelatin veronal buffer (GVB, Sigma-Aldrich, St. Louis, USA) supplemented with 13.3% ΔC1f2 serum and 16.6% CEMf2 was preincubated with or without inhibitors on ice for 15 min. 75µL of prechilled mixture was added to 25µL of GVB containing 5 × 105 CFUs of bacteria in microplate wells. Plates were incubated for 30 min at 37°C in 5% CO2 with shaking and then placed on ice to stop complement deposition. After bacteria were washed with ice-cold GVB, surface bound C3 or C4b/C4c bound were stained using murine anti-human C3 mAb (ThermoFisher Scientific, Waltham, USA; LF-MA0132, clone 28A1) and PE-conjugated anti-mouse Ab (BD Biosciences, Franklin Lakes, USA), or FITC-conjugated murine anti-human C4 mAb (ThermoFisher Scientific, Walktham, USA), and detected by flow cytometry using BD Accuri C6 Plus (BD Biosciences, Franklin Lakes, USA) and FCS Express software (Pasadena, USA).

### Detection of Opsonophagocytic Killing (OPK)

OPK assays were performed as previously described (17). Briefly, 7µg/mL of affinity-purified rFicolin-2 diluted in OBB was mixed 1:1:1 with OBB supplemented with 24% ΔC1f2 serum (normal or heat-inactivated), and with OBB alone or OBB with inhibitors. Following incubation on ice, 30µL of prechilled mixture was added to microwells containing 1x103 CFU bacteria suspended in 10µL of OBB. After 15 min incubation with shaking at 37°C, 40µL of OBB containing 4x105 HL60 cells (American Type Culture Collection, Manassas, USA, CCL-240) differentiated with DMF, was added to each well. Plates were incubated with shaking at 37°C for 30 min. Finally, 10uL from each microwell was spotted on THY agar plates, which were incubated overnight at 37°C with 5% CO2. CFUs were enumerated using ProtoCOL colony-counting software (Synbiosis, Cambridge, UK), in accordance with the well-characterized UAB OPK protocol (described at: http://www.vaccine.uab.edu. Accessed 2 July 2021).

### Whole Genome Sequencing

Genomic DNA extracted from strains MNY31, MNY32, MNY33, MNZ2293-A and MNZ2293-C using Monarch Genomic DNA purification kit (New England Biolabs, Ipswitch, USA) served as templates to construct DNA libraries with Nextera XT DNA sample preparation kit (Illumina, San Diego, USA). Sequencing was performed by the UAB Heflin Center Genomics Core Lab using the MiSeq platform (Illumina, San Diego, USA). Reads had adapters removed with Trimmomatic v0.38 (31), were assembled into draft genomes using *de-novo* assembler Unicycler v0.4.7 (32). Raw reads and assembled contigs are available on NCBI under Bioproject PRJNA779043. Scaffolds.fasta files were used for downstream analysis.

### Clonality Analysis

For clonality analyses, 39 additional pneumococcal serotype 11A genome assemblies from prior studies (**Table S1**) (33, 34), which represent strains obtained from 18 individuals, were obtained from NCBI. Species identity of the 39 NCBI genomes and five genomes from the current study, was confirmed using the ANIm method from pyANI v0.2.7 (35) and the cutoff of ANIm≥96% compared to a serotype 11A the reference genome (GenBank accession no. CP018838). Prokka v1.13.78 was run on all scaffold files to enumerate and identify open reading frames >500 bp in length. To identify core and accessory genomes (determined according to 95% nucleotide identity), we used.gff files produced by Prokka and Roary v3.13 (36) with the script “roary -e -n -p [number of genomes] -i 95 *.gff”. Using the “core_gene_alignment.fasta” as input, Snp-sites v2.4.0 (37) was used to remove indels and create multiFASTA alignment containing the core genome single nucleotide polymorphism (SNP) sites for each core genome. We calculated pairwise core genome SNP distance (from snp-sites multiFASTA alignment) and total genome average nucleotide identity (using pyANI results). The relatedness of strains obtained from the same patient served as references to empirically determine a cutoff defining clonal relationships between isolates, according to histogram visualization.

## Results

### Ficolin-2 Specifically Binds *wcjE-*Dependent Serogroup 11 Capsule Polysaccharide, But Not Broadly-Conserved Pneumococcal Surface Structures

**Figure 1:**
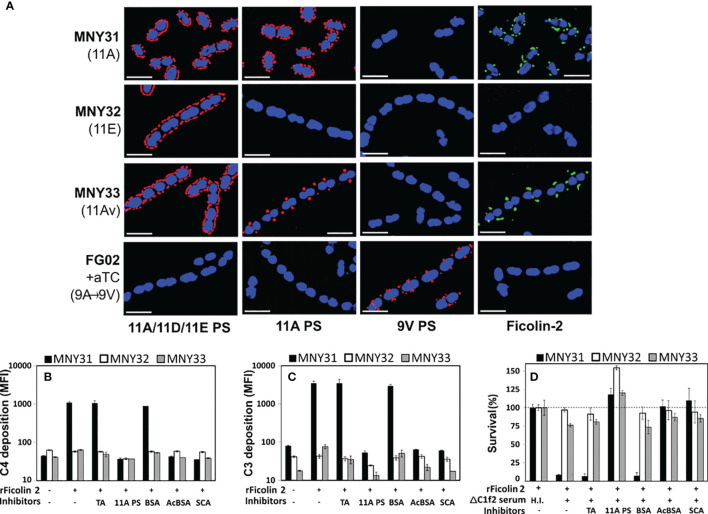
Ficolin-2 specifically recognizes pneumococcal serotype 11A capsule polysaccharide (PS) and triggers lectin pathway-mediated opsonophagocytosis. **(A)** SIM fluorescent micrographs showing DRAQ5-stained nuclei (blue) and the surface location of either capsule antigens (first three columns, red) or bound ficolin-2 on bacteria incubated in 5% normal human serum (fourth column, green). Y-axis lists strains with respective capsule serotypes in parentheses, and X-axis lists detected structures. White scale bars depict 2µm. **(B, C)** Deposition of complement components C4 (panel B) and C3 (panel C) on MNY31 (black bars), MNY32 (white bars), and MNY33 (gray bars) incubated in C1Q/ficolin-2-depleted (ΔC1f2) serum, with or without recombinant ficolin-2 (rFicolin-2) and potential inhibitors (represented on X-axis tables). Surface bound complement is represented as log mean fluorescent intensity (MFI) measured by flow cytometry. **(D)** Survival of the aforementioned strains in an opsonophagocytic assay, represented as a percentage of the average bacterial load when heat-inactivated (H.I.) ΔC1f2 serum was used (first bar set). Panels B-D show the average value of three biological replicates in a single experiment representative of at least two separate experiments. Error bars depict two-fold standard deviations. Identity of inhibitors is shown on the bottom row of X-axis tables. aTC, anhydrous tetracycline; TA, teichoic acid; BSA, bovine serum albumin; acBCA, acetylated BSA; SCA, silica clot activator.

**Figure 2:**
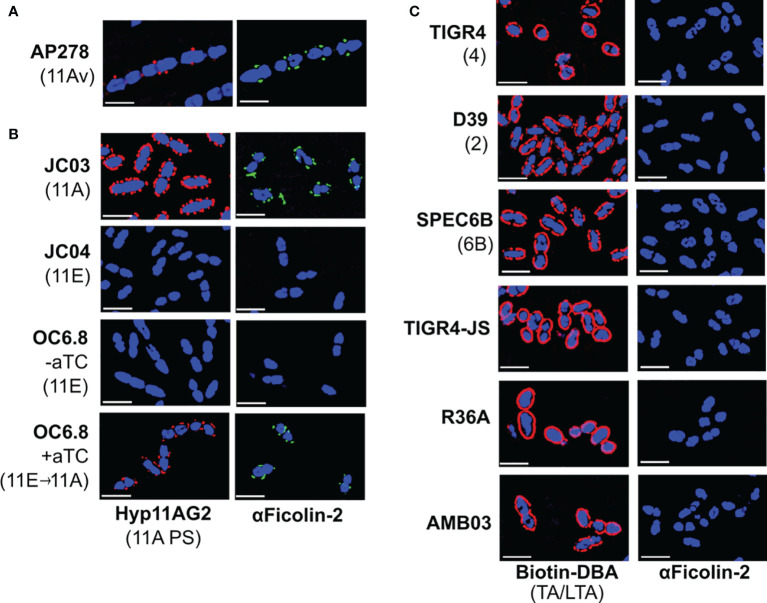
Ficolin-2 specifically binds to serotype 11A capsule polysaccharide (PS) and demonstrates no appreciable binding to other pneumococcal surface structures. **(A-C)** SIM fluorescent micrographs showing DRAQ5-stained nuclei (blue) and the surface location of either 11A capsule antigens (panels **A**, **B**, first column, red), Forssman antigen (ag) on pneumococcal teichoic/lipoteichoic acid (TA/LTA) (panel **C**, first column, red), or bound ficolin-2 on bacteria incubated in 5% normal human serum (panels **A-C**, second column, green). Y-axis lists strains with respective capsule serotypes in parentheses, and X-axis lists detected structures. White scale bars depict 2µm. aTC, anhydrous tetracycline.

To clarify the role of the LP in innate immunity against pneumococcal disease, we evaluated ficolin-2’s interaction with pneumococcal surface structures using fluorescent microscopy. We examined three closely related serogroup 11 strains: MNY31, whose surface is extensively decorated with *wcjE*-mediated 11A PS (detected with Hyp11AG2 mAb); MNY32, an 11E strain lacking 11A PS despite comparable expression of serogroup 11 capsule polysaccharide (detected with factor serum 11c); and MNY33, an 11A variant (11Av) expressing low levels of 11A PS that localized to the bacterial septa (**Figure 1A**). As expected, when bacteria were incubated with 5% human serum, ficolin-2 diffusely bound MNY31 and did not bind MNY32 (**Figure 1A**, fourth column). Notably, mirroring the focused distribution of 11A PS, ficolin-2 principally bound to the septa of MNY33. 11A PS and ficolin-2 ligands similarly colocalized to the septa of a different 11Av strain, AP278 (**Figure 2A**) (25).

We then evaluated whether *wcjE* mediates ficolin-2 binding through the modification of non-capsule surface structures by introducing an intact 11A *wcjE* allele under a tet promoter into multiple strains. As expected, exogenous *wcjE* expression in the 11E recombinant strain, OC6.8, was sufficient to restore 11A PS production and ficolin-2 binding (**Figure 2B**). In contrast, though exogenous *wcjE* expression was sufficient to mediate the synthesis of 9V PS (detected with Hyp9VG2 mAb) by a 9A recombinant strain, FG02, it did not result in ficolin-2 binding (**Figure 1A**). Lastly, confirming prior observations from flow cytometry and serum depletion assays (17, 18), we detected no binding of ficolin-2 to pneumococci expressing various non-serogroup 11 capsule types (i.e., TIGR4, D39, and SPEC6B, expressing serotypes 4, 2, and 6B capsule PS, respectively) or strains lacking capsule PS (i.e. TIGR-JS, R36A, and AMB03, with the latter being an 11A recombinant strain lacking essential capsule synthesis machinery but conserving an intact *wcjE*) (**Figure 2C**). This was despite all tested strains being bound by biotinylated Dolichos biflorus agglutinin (DBA), a lectin that binds the Forssmann antigen characteristically present in the non-reducing end of pneumococcal TA/LTA (30) (**Figure 2C**). Altogether, these findings confirm that ficolin-2 directly binds specific *wcjE*-dependent capsule PS structures.

### Partial or Complete Loss of *wcjE*-Mediated Serotype 11A Capsule O-Acetylation Results in Decreased LP-Mediated Complement Deposition and OPK

Serotype switching from serotype 11A to 11E through mutational inactivation of *wcjE* results in evasion of ficolin-2-mediated complement deposition and subsequent antibody-independent opsonophagocytic killing (OPK) (17). To investigate whether the partial reduction of *wcjE*-mediated 11A PS displayed by 11Av pneumococci (21) also eludes this innate defense, we first examined complement deposition on MNY31, MNY32, and MNY33 bacteria incubated in human serum depleted of both ficolin-2 and C1q, the key recognition molecule of the classical pathway of complement (23). Upon adding recombinant human ficolin-2 (rFicolin-2), we observed substantial C4 and C3 deposition on the 11A strain MNY31, with only minimal C3 deposition on the 11Av strain MNY33 and negligible deposition of either C4 or C3 on the 11E strain MNY32 (**Figures 1B, C**). This complement deposition was eliminated by preincubating rFicolin-2 with purified 11A capsule PS, silica clot activator (SCA), or acetylated bovine serum albumin (acBSA), which are all established inhibitors of ficolin-2 binding (17, 38), but not with pneumococcal TA or non-acetylated BSA. Lastly, consistent with prior findings (17), we observed high degree of *in vitro* ficolin-2-dependent, C1q-independent OPK of MNY31 (<10% of survival), greatly reduced ficolin-2-mediated OPK of MNY33 (~75% survival), but no significant killing of MNY32 (**Figure 1D**). In all cases, OPK was abrogated with heat inactivation of serum or preincubating rFicolin2 with binding inhibitors (**Figure 1D**). This demonstrates that even partial loss of 11A capsule PS expression confers a survival benefit against ficolin-2-dependent OPK.

### Whole Genome Sequencing Confirms Clonal Serotype 11A Derivatives Co-Isolated From Individual Cases of Invasive Pneumococcal Disease

As a species, pneumococci rely on commensal colonization of the human nasopharynx (NP), a host niche where serum lectins like ficolin-2 have a minimal presence. While 11A is among the most epidemiologically prevalent pneumococcal serotypes identified in NP carriage studies, it demonstrates the lowest propensity among major serotypes to cause invasive pneumococcal disease (IPD) (17). In contrast, the 11E/11Av phenotypes are almost exclusively identified among isolates causing disease, especially IPD (26), and 11E/11Av isolates harbor a wide variety of *wcjE* mutations that are rarely shared between two strains (19, 21, 25, 26). The disproportionate epidemiology of 11E/11Av and lack of clonally propagated *wcjE* mutations is consistent with these pneumococci encountering an environment favoring the convergent, “dead-end” microevolutionary inactivation of *wcjE* later in the infection process.

**Figure 3:**
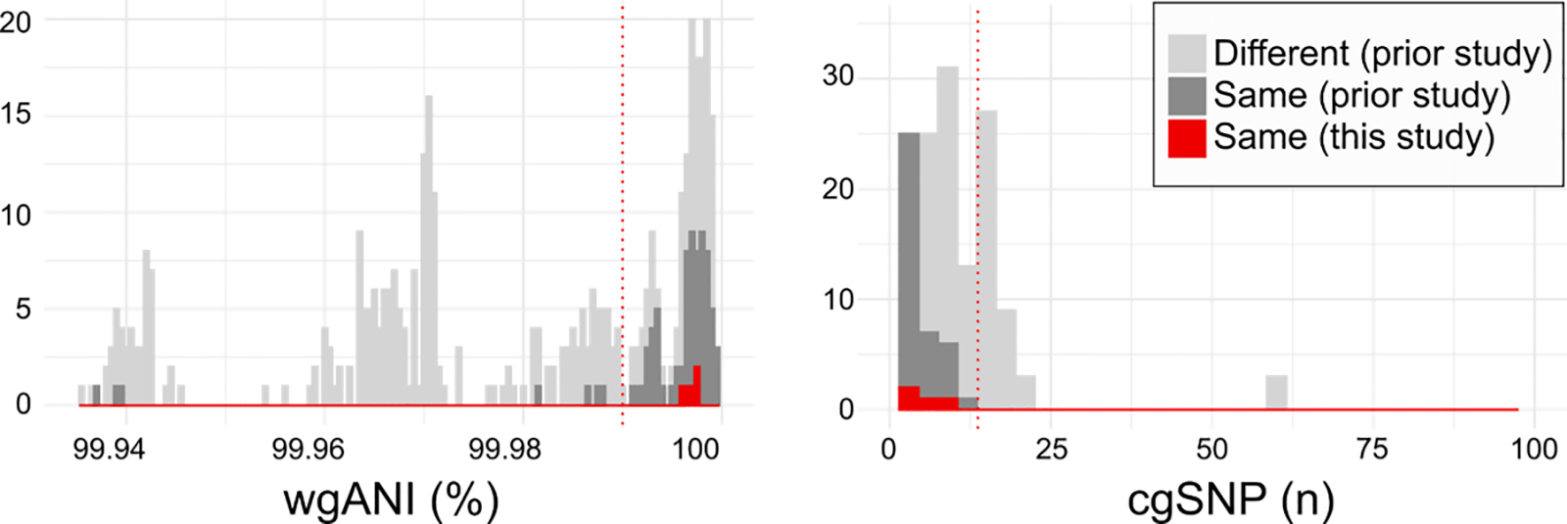
Relatedness of serogroup 11 isolates from current and prior studies. Histograms depicting the pairwise whole genome average nucleotide identity (wgANI, left panel) and core genome single nucleotide polymorphism distance (cgSNP, right panel) values between isolates obtained from different individuals (light gray), from the same individual in prior studies (dark gray) and the same individual in the current study (red). Dotted lines depict clonality cutoffs empirically-derived according to value distribution. For clarity, only extreme values are depicted.

Indeed, MNY31, MNY32, and MNY33, as well as the previously described 11A/11Av pair MNZ2293-A and MNZ2293-C (26), comprise groups of strains co-isolated from single blood cultures obtained in the USA and Israel, respectively. Whole genome sequencing and clonality analysis supports that these co-isolates originated from a single colonizing clone. The pairwise whole genome average nucleotide identity (wgANI) and core genome single nucleotide polymorphism distances (cgSNP) between MNY strains (wgANI=99.9972-99.9976% and cgSNP=4-6) and MNZ2293 strains (wgANI=99.9965% and cgSNP=8) were comparable to the values displayed by presumptive clonal strains belonging to serotype 11A lineages in prior studies (**Figure 3**) (33, 34).

MNY31 and MNZ2293-A each contain an intact and putatively functional *wcjE* (21). MNY32 and MNY33 contain an ISSpn5 transposon inserted at base pair 501 and an IS1515 transposon inserted at base pair 969 of *wcjE*, respectively. These insertions resulted in a five (MNY32) or three (MNY33) base pair direct repeat sequence flanking the respective insertion sequences. MNZ2293-C contains a C502T nonsense mutation in *wcjE*, as previously reported (26). Thus, these co-isolated strains likely represent instances in which *wcjE*-deficient variants (MNY32/MNY33 and MNZ2293-C) were in the process of replacing their respective 11A precursors (MNY31 and MNZ2293-A) during IPD.

## Discussion

**Figure 4:**
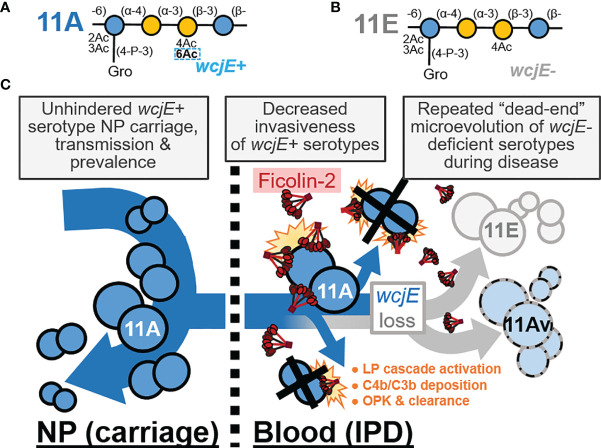
The innate, capsule-specific serological protection conferred by the ficolin-2 axis of the lectin complement pathway (LP). **(A, B)** The pneumococcal serotype 11A (panel **A**) and 11E (panel **B**) carbohydrate repeat units, which are polymerized into capsule polysaccharide. The dotted rectangle indicates the *wcjE-*dependent 6-O-acetylation (Ac) that mediates ficolin-2 recognition of 11A capsule. Blue and yellow circles represent glucose and galactose, respectively. Gro, glycerol; P, phosphate. **(C)** Epidemiological observations that support a model in which ficolin-2 protects against invasive pneumococcal disease (IPD) by transmissible 11A strains, while selecting for the emergence of non-transmitted 11E and 11Av clones. NP, nasopharynx; OPK, opsonophagocytic killing.

We propose that ficolin-2’s specific recognition of O-acetylated capsule glycopolymers (17) is the principal factor providing population-wide, innate serological protection against 11A IPD and spurring the convergent loss of capsular O-acetyltransferase genes across independent infections, while having negligible impact on 11A prevalence in NP carriage (**Figure 4**). This interaction does not appear to be limited to *wcjE*-associated serotypes. Pneumococcal serotype 35B capsule PS, which contains type-defining β-galactofuranose-2-O-acetylation mediated by another O-acetyltransferase gene, *wciG*, is also targeted by the ficolin-2 axis of the lectin pathway (39). Reminiscent of 11Av/11E strains, serotype 35D strains arise from 35B precursors as result of sporadic microevolutionary inactivation of *wciG*, are not recognized by ficolin-2, and are associated with IPD isolates (39–41). Furthermore, glycopolymer-specific reactivity is not unique to one axis of the LP. Akin to ficolin-2, ficolin-1 (also known as M-ficolin) is an activator of the LP in humans that preferentially binds pneumococcal serotypes 19B and 19C, but not other epidemiologically-relevant serotypes, including serotypes 19A and 19F (42).

The strong serotype-specific, lectin-capsule interactions described here are consistent with epidemiologic and *in vivo* evolutionary observations and likely reflect the components of ficolin-2 biology most relevant to combatting infections. We cannot fully exclude that ficolin-2 also has serotype-independent interactions with broadly-conserved pneumococcal features, as reported by others (9, 12). Indeed, the ficolin-2 carbohydrate-recognition domain contains multiple binding sites through which the molecule can interact with different ligands at varying degrees of affinity (43, 44), and our *in vitro* assays using 5% serum may not detect all interactions that occur under physiological conditions. However, as described above, population-wide studies do not support that the LP confers protection beyond glycopolymer-specific targets. Because there is a vast repertoire of microbial glycopolymers to which LP lectins could display some degree of *in vitro* affinity, it is critical to develop criteria to assess the immunological relevance of these interactions.

Regardless, these glycopolymer-specific interactions exemplify why LP deficiencies cannot be appropriately investigated in a serotype-naïve fashion. For example, the portion of infections caused by prevalent serotypes that do not effectively trigger the LP (i.e., serogroups 6, 14, 19A/F, etc.) (17) can mask the significant protection provided against infections from a narrower subset of LP-triggering serotypes (i.e., serogroup 11, 35, 19B/C, etc.). Notably, current pediatric pneumococcal conjugate vaccines (PCV) do not include serotypes 11A and 35B, and, as result of widespread PCV usage, these serotypes are now among the most prevalent in surveys of NP carriage (45). However, these serotypes continue to be rarely implicated in pediatric IPD (45), consistent with our LP-mediated protection model (**Figure 4**). A complete understanding of the host-microbe interactions that dictate human health necessitates the study of less pathogenic microbial populations whose “virulence” is hampered by their immediate recognition and clearance by innate immune factors. Therefore, future investigations should capitalize on the fact that PCV implementation, through enrichment of LP-triggering serotypes in pediatric and adult populations, is putatively creating an optimal epidemiological scenario to examine the impact of the LP deficiencies and decipher the role of these soluble lectins in preventing major human infections.

## Author’s Note

UAB has Intellectual Property Rights on some reagents used in the study. All the authors of this study are UAB employees.

## Data Availability Statement

The datasets presented in this study can be found in online repositories. The names of the repository/repositories and accession number(s) can be found below: https://www.ncbi.nlm.nih.gov/, PRJNA779043 (whole genome sequences) and MZ054181 (OC6.8 insert sequence).

## Ethics Statement

The studies involving human participants were reviewed and approved by University of Alabama in Birmingham School of Medicine Internal Review Board. The patients/participants provided their written informed consent to participate in this study.

## Author Contributions

MN: Supervised the group, devised and executed the project, had full access to all study data, and takes responsibility for the integrity and accuracy of the data analysis. JC: Devised and performed experiments, drafting and critical revision of the manuscript. JY and FG: Performed experiments, data analysis, and interpretation, critical revision of the manuscript. All authors contributed to the article and approved the submitted version.

## Funding

This work was supported with funding to MN from the National Institutes of Health (R01 AG050607) and funding to JJC from the NIH (K08 AI148582).

## Conflict of Interest

The authors declare that the research was conducted in the absence of any additional commercial or financial relationships that could be construed as a potential conflict of interest.

## Publisher’s Note

All claims expressed in this article are solely those of the authors and do not necessarily represent those of their affiliated organizations, or those of the publisher, the editors and the reviewers. Any product that may be evaluated in this article, or claim that may be made by its manufacturer, is not guaranteed or endorsed by the publisher.
