# Supplementary material for: Ficolin-2 Lectin Complement Pathway Mediates Capsule-Specific Innate Immunity Against Invasive Pneumococcal Disease
Source: Front Immunol. 2022 Mar 28;13:841062. doi: 10.3389/fimmu.2022.841062 (PMC8996173; doi:10.3389/fimmu.2022.841062)
Supplement: Supplementary file 1 [file Table_1.docx]

Supplementary Material

# Supplementary Figures and Tables

**Table S1. List of publicly available serotype 11A S. pneumoniae genomes included in comparative genomic analysis**
